# Potency-directed similarity searching using support vector machines

**DOI:** 10.1186/1758-2946-4-S1-P12

**Published:** 2012-05-01

**Authors:** Kathrin Heikamp, Anne M Wassermann, Jürgen Bajorath

**Affiliations:** 1 Department of Life Science Informatics, B-IT, LIMES Program Unit Chemical Biology and Medicinal Chemistry, Rheinische Friedrich-Wilhelms-Universität Bonn, Dahlmannstr. 2, D-53113 Bonn, Germany

## 

Searching for active compounds in screening databases is one of the main tasks in chemoinformatics [1,2]. For this purpose, different approaches have been developed, with an increasing interest in machine learning and data mining methods [3]. Among these, support vector machine (SVM) learning has proven to be a powerful search technique in many instances [3]. Several applications of SVMs have been reported that focus on compound ranking in similarity searching [4-6]. However, similarity search and machine learning methods that are commonly utilized for virtual screening generally do not take compound potency information into account. Regardless of the applied methods, one typically attempts to distinguish “active” from “inactive” compounds. With the exception of QSAR models adapted for compound screening, no approaches have thus far been introduced that incorporate potency information as a parameter and direct search calculations toward the recognition of potent hits. Here, an SVM approach for potency-directed virtual screening is introduced [7]. A newly designed structure-activity kernel and an SVM linear combination model take potency information of reference molecules into account. Applied to high-throughput screening data sets, this potency-directed SVM approach met or exceeded the recall performance of standard SVM ranking and led to a notable enrichment of highly potent hits in database selection sets.
